# The Integration of Stressful Life Experiences Scale and the Inventory of Complicated Spiritual Grief: The Italian Validation of Two Instruments for Meaning-Focused Assessments of Bereavement

**DOI:** 10.3390/bs11110149

**Published:** 2021-10-29

**Authors:** Robert A. Neimeyer, Ines Testoni, Lucia Ronconi, Gianmarco Biancalani, Marco Antonellini, Laura Dal Corso

**Affiliations:** 1 Department of Psychology, University of Memphis, Memphis, TN 38111, USA; neimeyer@memphis.edu; 2 Portland Institute for Loss and Transition, Portland, OR 97209, USA; 3 Department of Philosophy, Sociology, Education and Applied Psychology (FISPPA), University of Padua, 35131 Padua, Italy; gianmarco.biancalani@unipd.it (G.B.); marcoantonellini@gmail.com (M.A.); dalcorso@unipd.it (L.D.C.); 4 Emili Sagol Creative Arts Therapies Research Center, University of Haifa, Haifa 3498838, Israel; 5 IT and Statistical Services, Multifunctional Centre of Psychology, University of Padua, 35131 Padua, Italy; l.ronconi@unipd.it

**Keywords:** Integration of Stressful Life Experiences Scale (ISLES), Inventory of Complicated Spiritual Grief (ICSG), Italian validation, meaning making in bereavement

## Abstract

Background: Bereavement is an inevitable event that can cause pain, discomfort, and negative consequences in daily life. Spirituality and religiosity can help people cope with loss and bereavement. Sometimes, however, the death of a loved one can challenge core religious beliefs and faith, which has been found to be a risk factor for prolonged mourning. Objectives: (1) Determine whether the Italian versions of the Integration of Stressful Life Experiences Scale (ISLES) and Inventory of Complicated Spiritual Grief (ICSG) are valid in translation; (2) Evaluate the impact of socio-demographic variables on ISLES and ICSG dimensions; (3) Test whether Complicated Spiritual Grief mediates the relation between meaning reconstruction after loss and integration of the loss experience; (4) Test whether the representation of death as a form of passage or annihilation further moderated the relation between Complicated Spiritual Grief and integration of the loss. Methods: The sample is composed of 348 participants who had lost a loved person in the prior two years. Results: The ISLES and ICSG were validated in Italian and are more appropriately interpreted as having a unifactorial structure. A greater spiritual crisis was manifested in participants with less education, who did not actively participate in religious life, and who had lost a friend rather than a close relative. As hypothesised, spiritual struggle in grief mediated the role of continuing bonds, Emptiness and Meaninglessness, and Sense of Peace in predicting integration of the loss. Furthermore, death representation moderated the impact of spiritual grief on loss, such that those participants who viewed death as a form of annihilation rather than passage reported greater integration of the loss. Conclusion: The role of meaning making in integrating significant loss is partly accounted for by spiritual struggle in a way that can be analysed in Italian contexts through the use of these newly validated instruments.

## 1. Background

Grief is both a natural and inevitable process and a constructed event resulting from a permanent or temporary disruption, separation, loss, or change in a relationship over which the individual has no control. The loss of an intimate attachment relationship through death poses profound challenges to our adaptation as living beings. On the one hand, core characteristics of our response to loss reveal our evolution as biological and social beings, arising from the disruption of attachment bonds necessary for survival. On the other hand, individuals respond to loss at symbolic as well as biological levels, ascribing significance to the symptoms of separation experienced and to the changes in social relationships caused by the death of a beloved person [1,2]. A major loss, such as the disappearance or death of a loved one, can cause severe grief and carries important consequences for daily life [3,4,5]. Mourners experience and express grief uniquely and differently from each other, based on their cultural and family background, life experiences, personal values, and deepest beliefs [6]. Indeed, human beings seek meaning in mourning and do so by trying to provide a coherent sense to their suffering to preserve a sense of continuity with whom they have lost while at the same time integrating the new reality into their changing world. From a constructivist perspective, mourners commonly find themselves reflecting on the meaning of death, especially when they are unprepared to deal with this topic and have to find new significance in their sense of life after loss [7].

Although the death of a beloved person can be horrific for most mourners, some individuals are more susceptible to developing complicated grief responses than others. One potential vulnerability factor predisposing to such reactions may be related to the models developed from a person’s experience with primary attachment figures, most notably in one’s relationship to parents [1]. The work of re-signifying the existential experience is extremely challenging [8], and just when these people most need to maintain a mutually supportive relationship with others, they may isolate themselves as they deal with the change of their internal models of attachment and their core beliefs, on which a feeling of security is based [9]. In addition, decisive is the contextualisation of the loss experience: for example, difficulty in processing the loss of a child [10] or the manner in which the loss occurred [11]. However, many other risk factors have been identified in recent research, including fewer years of education, depression, anxiety, poor physical health, maladaptive dependency, and attachment traits, lower perceived social support, family conflict at end of life, family difficulty accepting death, younger age, fear of death, and place of death [12]. In contrast, adaptive meaning making in loss is fostered by supportive interventions that use narrative techniques [3] as well as grief counselling for mourners struggling with intense symptoms [13,14]. Grief counselling helps mourners cope with the negative effects of loss, i.e., physical, emotional, social, spiritual, and cognitive reactions [15]. Grief counselling involves the personal reconstruction of a world of meaning that has been challenged by loss [16], a process that can be co-constructed in the safe environment of a therapeutic setting.

In a meaning-focused grief counselling perspective, the entire universe of the mourner’s beliefs and convictions is taken into account. Religious belief versus non-belief can make an essential difference in the mourner’s interpretation of death, as can the confession of faith professed [17]. All these elements have to be considered for the planning of a specific and person-centred intervention.

The literature has long confirmed that spirituality and religiosity can help people cope with stressful situations and also overcome grief [18]. The literature shows how spirituality and religiosity can promote an understanding of the human condition, the achievement of a state of wholeness, connection with oneself, others, nature, and God/life forces, and finding meaning and purpose in life even in the most difficult circumstances [19]. A religious coping strategy helps mourners find comfort in believing that the deceased is in a better place and that when they die, they will be reunited with their beloved [20].

However, Neimeyer et al. [18] have found that the death of a loved one can also trigger spiritual struggle. Many studies have suggested that for some mourners who are spiritually inclined, bereavement can cause a crisis of faith that calls into question their long-held religious beliefs. This kind of prolonged and debilitating spiritual distress, which has been termed Complicated Spiritual Grief (CSG), includes the collapse or erosion of the griever’s sense of security with God and/or their faith community and has shown an empirically consistent association with complicated grief [21].

## 2. The Research

### Aims and Research Questions

Research related to grief and loss in recent years has benefited from the development and validation of scales to both test important theoretical propositions and contribute to the assessment of client resources and vulnerabilities in the clinical context. In Italy, some basic clinical instruments have been translated and validated for assessing responses to bereavement, such as the *Inventory of Complicated Grief* (ICG) by Prigerson et al. [22], used internationally to assess the intensity of complicated grief and validated in Italy by Carmassi et al. [23]; the *Continuing Bonds Scale* by Field and Filanosky [24], validated by De Luca et al. [25]; and the *Prolonged Grief Disorder* (PG-13) by Prigerson et al. [26], validated by De Luca et al. [27]. In Italy, a meaning-focused approach to bereavement is receiving increasing attention, but there is a lack of useful tools to study this approach and to promote its incorporation into practice settings. With the recent development of training courses that focus on the acquisition of these skills [28], the present study was conducted to translate and validate in the Italian context scales developed to assess the extent to which mourners could construct meaning in loss. The main aims are as follows:

1. Determine whether the Italian versions of the Integration of Stressful Life Experiences Scale (ISLES) and the Inventory of Complicated Spiritual Grief (ICSG) are reliable and valid, as reflected by their convergence with related measures;
2. Evaluate the impact of socio-demographic variables on the ISLES and ICSG;
3. Test whether the relation between meaning reconstruction after loss and the degree of integration of the loss into the mourner’s meaning system is mediated by spiritual struggle;
4. Test whether the representation of death as a form of passage or annihilation moderates the relation between spiritual struggle and the integration of the loss experience.

## 3. Methods

### 3.1. Participants

The study was carried out with the involvement of hospices and funeral homes in Northern and Central Italy. People interested in the research were sent the link to the questionnaire, including informed consent. Only Italian speakers over 18 who had suffered the loss of a loved one in the previous two years were selected. The questionnaire was anonymous and uploaded to the LimeSurvey platform. The link was received by 488 people, 348 of whom decided to participate, all of them meeting the inclusion criteria. Their ages ranged between 18 and 91 (M = 46; DS = 19), of whom 238 (68%) were women and 110 (32%) were men. The participants’ education levels were high: 53% had a university degree or doctorate, 35% had a secondary school diploma, and 12% had completed middle school. Regarding their losses, 27% grieved a parent, 23% a grandparent, 14% a close friend, and 36% another relative or close person (7% spouses, 5% children, 6% brothers, 9% uncles/aunts, 3% nephews, 2% cousins, and 4% other people). Concerning marital status, 41% were married, 28% single, 17% engaged, 8% widowed, and 6% other (separated, divorced, other). Regarding religion, 78% were Catholic, 19% atheist, and 3% other (Orthodox Christian, Muslim, Buddhist). Of these, 65% were non-practising believers.

### 3.2. Procedure

The translation of the instruments into Italian and back-translation into English were carried out by a team of 2 translators, who resolved minor disagreements through consensus following recommended procedures [29,30]. Recruitment of the sample occurred by requesting the help of hospices and funeral homes, which offered participation in the study to families they had served over the last two years. The subjects who responded to the announcement did so by indicating an e-mail address at which they could be contacted. The address was entered into the LimeSurvey system of the University of Padua so that the link to access the questionnaires could be sent to them. The link was not universal but personal so that it could be used only once, ensuring that only a specific person filled out the questionnaires. The questionnaires were offered online with an average completion time of approximately 20 min, and the data were collected before the COVID-19 pandemic.

### 3.3. Ethical Considerations

The research followed APA Ethical Principles of Psychologists, the APA Code of Conduct, and the principles of the Declaration of Helsinki, and all research objectives and the methodology of analysis were explained to the participants. The Ethics Committee of the University of Padua approved the study (n. 38617F829E394F486DB1549F8130C1C6).

### 3.4. Measures

*The Grief and Meaning Reconstruction Inventory* (GMRI; [31]) measures the type of meaning attributed to the loss and the form that the meaning itself acquires (e.g., “I’ve come to understand that life is short and that it offers no guarantees”). It includes 29 items with a Likert 5-point response scale (1 = strongly disagree; 5 = strongly agree). The authors of the original English version of the GMRI detected a five-factor structure: Continuing Bonds (CB), Personal Growth (PG), Sense of Peace (SoP), Emptiness and Meaninglessness (EM), and Valuing of Life (VL). The Cronbach’s alpha of the original total scale is 0.84 and for the five factors, respectively is: 0.85, 0.83, 0.79, 0.76, and 0.76. For this study, we have used the scale validated in Italian [32], and the current Cronbach’s alphas are 0.76 for the total scale and for the five factors, respectively: 0.71, 0.80, 0.77, 0.70, and 0.61.

*The Integration of Stressful Life Experiences Scale* (ISLES; [33]): assesses the degree to which a stressful life experience has been adaptively incorporated into a larger life story that can promote a sense of internal coherence and foster a confident and promising vision of one’s future (example item “Since this event happened, I don’t know what I will do with my life in the future”). It consists of 16 items with a 5-point Likert response scale (1 = strongly disagree; 5 = strongly agree). The authors of the original English version detected a two-factor structure for the scale: “Footing in the World” and “Comprehensibility”. The Cronbach’s alpha of the original total scale is 0.94 and of the two factors, respectively is 0.94 and 0.85. For this study, Cronbach’s alpha of the total scale is 0.92 and of the two factors, respectively, is: 0.91 and 0.80.

*The Inventory of Complicated Spiritual Grief* (ICSG; [21]) measures the respondent’s degree of spiritual struggle or crisis related to their relationship with either God or fellow worshippers (item example “I no longer feel safe and protected by God”). It consists of 18 items with a 5-point Likert response scale (0 = not at all true; 4 = absolutely true). The authors detected a two-factor structure: “Insecurity with God” and “Disruption in religious practice”. The Cronbach’s alpha of the original total scale is 0.96. For this study, Cronbach’s alpha of the total scale is 0.94.

*The Testoni Death Representation Scale* (TDRS; [34]) investigates ontological representations of death as a form of passage or annihilation (example item “Death is just a passage. After my death, I will continue to exist and remember the experiences of this life”). It consisted of six items with a 5-point Likert response scale (1 = totally disagree; 5 = totally agree). Factor analysis revealed a single-factor structure, termed “death representation”. Higher values indicate the representation of death as annihilation. The original scale was validated with an Italian sample with a Cronbach’s alpha of 0.86. For this study, Cronbach’s alpha of the total scale is 0.84.

### 3.5. Data Analyses

#### 3.5.1. Validation of the Instruments

First, we performed an analysis to validate the two scales newly translated into Italian and used for the first time in the study: the ICSG and ISLES. We used Confirmatory Factor Analysis (CFA) for each scale, comparing the fit of two models, a one-factor model, and a multifactor model, according to the factorial structure presented in the original versions. We considered several fit indices to evaluate the models: chi-square, Comparative Fit Index (CFI), Tucker–Lewis Index (TLI), and Root Mean Square Error of Approximation (RMSEA). Cut-off values for adequate fit for CFI and TLI were >0.90 [35] and for RMSEA < 0.08 [36]. Moreover, to assess the fit of nested models, we adopted the delta CFI criterion. Cheung and Rensvold [37] recommended that if the CFI difference between two nested models is smaller than 0.01, the hypothesis of no difference in fit between the two competing models should not be rejected. We decided to use the delta CFI instead of the delta chi-square because it is less sensitive to sample size. We also calculated the descriptive statistics and correlations between scales and Cronbach’s alpha for all factors.

#### 3.5.2. Impact of Socio-Demographic Variables on ICSG and ISLES

Next, we evaluated the impact of socio-demographic variables on the total scores of the ICSG and ISLES using Analysis of Covariance (ANCOVA), including sex, education (undergraduate vs. graduate/postgraduate), marital status (non-engaged vs. engaged), religious practice (non-practising believer vs. practising believer), and relationship with the deceased person as between-subject factors. This last factor was divided into three degrees (1 = nuclear family member, including parent, spouse, and child; 2 = other family member; 3 = friend or other person). In all analyses, age was treated as a covariate.

#### 3.5.3. Path Model

A path model was calculated to evaluate the mediating role of spiritual struggle (as assessed by the ICSG) in the relationship between meaning reconstruction factors (as assessed by the GMRI factors CB, PG, SoP, EM, VL) on the one hand and the ability to integrate the death meaningfully (as assessed by the ISLES) on the other. In addition, death representation was evaluated as a possible moderator in this relationship.

Analyses were carried out using SPSS 26 (IBM Corp. Released 2016. IBM SPSS Statistics for Windows, Version 26.0. Armonk, NY) for descriptive, reliability, and the correlation and R package lavaan for CFA and path analysis (Rosseel, 2021) [38].

## 4. Results

### 4.1. Validation of the Instruments

The two-factor model for ICSG and ISLES showed suitable fit indices, but the one-factor model was preferred (Table 1). Although delta chi-square was always significant, indicating a better fit of the model with two factors than one-factor, Delta CFI between the two models is never greater than |0.01| (Table 1), indicating the preference for the more parsimonious one-factor model. Therefore, we decided to focus on the total score for both scales for subsequent path analysis. The reliability of all GMRI factors is adequate, with Cronbach’s alpha values between 0.70 and 0.80 except for VL (alpha = 0.61). The reliability is high for all other scales (TDRS, ICSG, and ISLES) with alpha values greater than 0.80.

The descriptive statistics for the scales are shown in Table 2. We can observe that the GMRI factors with higher average scores, i.e., above the middle point of the 5-point Likert scale (3) are CB, PG and VL (respectively *t* = 25.49 df = 347 *p* < 0.001, *t* = 2.91 df = 347 *p* = 0.004 and *t* = 16.87 df = 347 *p* < 0.001). The EM factor has an average score lower than the midpoint (*t* = −7.81 df = 347, *p* < 0.001), while the SoP factor has an average score that does not differ from the midpoint (*t* = 1.46 df = 347 *p* = 0.145). For TDRS, no difference from the midpoint (18) was observed (*t* = 1.26 df = 347 *p* = 0.210), which indicates the presence of a non-radical position of participants on the representation of death from a passage to total annihilation. A significantly lower average score under midpoint (36) was found for ICSG (*t* = −13.59 df = 347 *p* < 0.001), indicating lower levels of spiritual struggle in the sample. Finally, a significantly higher average score above the midpoint (48) was found for ISLES (*t* = 21.74 df = 347 *p* < 0.001), indicating the presence of a generally suitable ability to integrate the loss experience into participants’ meaning systems.

The ability to integrate the loss as measured by the ISLES displayed a moderate negative correlation with Complicated Spiritual Grief on the ICSG, a strong negative correlation with the Emptiness and Meaninglessness (EM) factor of the GMRI, and a moderate positive correlation with the Sense of Peace (SoP) factor of GMRI. Spiritual struggle as assessed by the ICSG correlates with death representation as annihilation and correlates negatively with the Continuing Bond (CB) and Sense of Peace (SoP) factors of the GMRI and positively with Emptiness and Meaninglessness (EM). Finally, death representation as annihilation shows a moderate negative correlation with the CB factor of GMRI.

### 4.2. Impact of Socio-Demographic Variables on ICSG and ISLES

An ANCOVA for the ICSG, with the socio-demographic variables and the relationship with the deceased person included as between-subject factors and age included as a covariate, showed a significant effect of education F(1337) = 10.24 *p* = 0.002 η_p_^2^ = 0.03; religious practice F(1337) = 83.17 *p* < 0.001 η_p_^2^ = 0.20; and the relationship to the deceased F(1337) = 3.96 *p* = 0.020 η_p_^2^ = 0.02. No significant effects of sex, F(1337) = 1.43 *p* = 0.232 η_p_^2^ = 0.03; age F(1337) = 2.81 *p* = 0.095 η_p_^2^ = 0.01; or marital status F(1337) = 1.97 *p* = 0.162 η_p_^2^ = 0.01 were found. In particular, adjusted mean values showed higher scores on ICSG for those with less education (M = 24.09 SE = 1.40 vs. M = 18.58 SE = 1.33), for non-religious practitioners vs. religious practitioners (M = 29.68 SE = 1.18 vs. M = 12.99 SE = 1.59), for those who had lost a friend or another person vs. who had lost a family member, whether close or not (respectively, M = 25.36 SE = 2.20 for friend/other, M = 20.55 SE = 1.50 for very close family member and M = 18.10 SE = 1.39 for other family members).

An ANCOVA for the ISLES, with the socio-demographic variables and the relationship to the deceased person included as between-subject factors and age included as a covariate showed a significant effect of sex, F(1337) = 15.95 *p* < 0.001 η_p_^2^ = 0.05; education F(1337) = 10.17 *p* = 0.002 η_p_^2^ = 0.03; and marital status F(1337) = 6.79 *p* = 0.010 η_p_^2^ = 0.02. No significant effects of age F(1337) = 0.04 *p* = 0.844 η_p_^2^ < 0.01; religious practice F(1337) = 3.11 *p* = 0.079 η_p_^2^ = 0.01; and the relationship to the deceased F(2337) = 1.38 *p* = 0.253 η_p_^2^ = 0.01 were found. In particular, adjusted mean values showed higher scores on the ISLES for men than for women (M = 65.32 SE = 1.15 vs. M = 60.14 SE = 0.81), for those with more education (M = 64.69 SE = 0.95 vs. M = 60.78 SE = 1.00), and for those engaged in a relationship rather than single (M = 64.36 SE = 0.90 vs. M = 61.11, SE = 1.05).

### 4.3. Path Model

**Figure 1:**
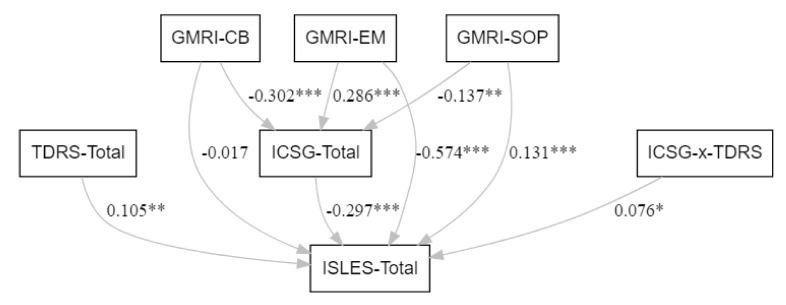
Path model (standardised coefficients; * *p* < 0.05, ** *p*< 0.01, *** *p* < 0.001).

A path analysis was conducted to test the mediating role of ICSG in accounting for the relation between GMRI factors on the one hand and the ISLES on the other, as well as the moderating role of the TDRS in the relation between the ICSG and ISLES. The initial model included direct and indirect effects (through ICSG) of all GMRI factors on ISLES, but the final model does not include two factors of the GMRI, PG, and VL, as these were without significant linkages to either the mediator or dependent variable (Figure 1). The model explains 19% of the variance in the ICSG and 59% of the variance in ISLES.

The mediation of meaning making on loss integration by the ICSG was confirmed; as the three indirect effects of GMRI factors on ISLES are statistically significant (respectively beta = 0.09 *p* < 0.001 for CB, beta = 0.04 *p* = 0.012 for SOP and beta = −0.08 *p* < 0.001 for EM). In particular, the indirect effect is positive for CB and SoP, i.e., higher levels of continuing bonds with the loved one and a Sense of Peace regarding the death are associated with greater integration of the loss and, in contrast, is negative for Emptiness and Meaninglessness (EM), i.e., higher levels are associated with less integration of the loss experience. For SoP and EM, there are also significant direct effects on the ISLES (respectively beta = 0.13 *p* < 0.001, beta = −0.57 *p* < 0.001), indicating that only part of the shared variance between these factors and ISLES is explained by their relationship with ICSG. However, the effect of CB on the integration of the loss was fully explained by the ICSG.

As hypothesised, death representation as assessed by the TDRS moderated these effects; the interaction between the ICSG and TDRS is statistically significant (beta = 0.08 *p* = 0.027). Slope analysis evidences a more negative impact of ICSG on ISLES for subjects with lower (lower than 1 SD from mean) scores than for subjects with higher scores (higher than 1 SD from mean) on the TDRS (respectively, beta = −0.66 *p* < 0.001 and beta = −0.52 *p* < 0.001), i.e., spiritual struggle on the ICSG is more strongly associated with the integration of the loss for those subjects who viewed death as a passage rather than as annihilation. Finally, the TDRS also showed a significant and positive effect on ISLES (beta = 0.11 *p* = 0.006), indicating higher death representation as annihilation is associated with greater integration of the loss experience.

## 5. Discussion

The analyses of the results allow us to state that all the objectives have been achieved. For the first aim, the ISLES and the ICSG were validated in Italian (see Appendix A). Although the original two-factor structure had an adequate fit, model comparison indices indicated that the single-factor structure was better, so this model was adopted in the present study, where greater parsimony is desirable in conducting a path analysis. First, the ISLES in its Italian translation assesses the degree to which a salient life stressor, in this case, the death of a loved one, has been adaptively incorporated into one’s global meaning system. Its usefulness in the original English has already been confirmed by the literature, which has found it to be a robust predictor of mental and physical health [39,40]. Second, the Italian ICSG is an easy-to-use scale for assessing spiritual crisis during bereavement that can be used in a variety of clinical settings, helping therapists determine which religious/spiritual issues may be salient while the believer is working through the bereavement. Indeed, as many therapists hesitate to inquire into religious issues and such hesitation or cautiousness can limit the utility of their dialogue, the ICSG may prove useful not only for checking the presence of this problem but also as a “conversation starter” on the impact of the loss on their relationship with God or with their community of spiritual practice [32].

The strength of the mourner’s Continuing Bond with the deceased was positively correlated with personal growth, valuing life, and perceiving death as a passage rather than annihilation, but also with Emptiness and Meaninglessness and spiritual struggle, possibly reflecting their acute grief over the loved one’s death. This first result confirms what previous literature has shown, i.e., that the representation of death as a passage is related to a positive representation of spirituality and helps people deal with stressful circumstances [41,42,43]. In the same direction, personal growth was positively correlated with VL, and this result is in line with literature on post-traumatic growth, which documents the greater appreciation of living reported by survivors of adversity who report developing through the experience [44]. Relatedly, the Sense of Peace about the death was inversely correlated with spiritual crisis and positively with the integration of the loss as a meaningful experience. In contrast, Emptiness and Meaninglessness positively correlated with spiritual crisis and negatively correlated with the integration of loss. Finally, spiritual crisis was inversely correlated with the ability to integrate the loss experience, and, similarly to previous studies, the representation of death as an absolute annihilation was positively correlated with spiritual crisis [45].

For the ICSG, those with lower education, less religious practice, and those who had lost a friend or another person rather than a family member reported greater spiritual struggle. As widely considered by the literature [46], given that members of religious communities often cultivate extensive friendships, it may be particularly painful to lose a friend for people who do not belong to a community. Finally, men, those with higher education, and those who were engaged in a relationship showed a higher ability to integrate the loss.

Testing the mediating role of spiritual struggle in the relation between meaning reconstruction after grief and the integration of the loss, the effect was confirmed for three factors of the GMRI: CB, EM, and SoP.

For SoP and CB, the indirect effect through ICSG is positive. This means that mourners who experience a greater Sense of Peace and cultivate a stronger bond with the deceased enter less into a spiritual crisis because of the death of a loved one, and this helps them to integrate the experience of mourning more easily. This result is in line with much of the literature on CB (e.g., the work of [47]) and the literature showing the positive effect of representing death as a passage [48]. In contrast, with EM, the indirect effect is negative, meaning that the more people attribute a sense of Emptiness and Meaninglessness to the loss, the greater the spiritual crisis they experience, and this leads to greater difficulty in integrating the loss. Again, this confirms what has been widely discussed in the literature (i.e., the work of [49]).

Finally, testing the moderating effect of death representation on the relation between CSG and the integration of the loss experience produced statistically significant results. This means that the negative impact of spiritual crisis following the loss of a loved one on the integration of the event itself is greater among those who portray death as a passage.

## 6. Conclusions

The results of this study show that the Italian translations of the Integration of Stressful Life Experiences Scale and Inventory of Complicated Spiritual Grief are useful tools to evaluate the spiritual condition of mourners and their ability to integrate the experience of loss. Bereavement studies are progressively developing an evidence base to inform those implementing targeted interventions to support mourners. The results of our study suggest the utility for therapists of using these tools after suitable training. Spirituality and religiosity help attribute meaning to loss, especially when the representation of death as a passage rather than total annihilation is associated with the Continuing Bond dimension. We hope the introduction of these tools in an Italian context invites greater attention to the role of meaning making and spiritual struggle in bereavement, both on the part of the research and practice communities.

### Research Limitations and Future Developments

The cross-sectional nature of this study and its lack of a multi-method approach can be overcome by adopting a longitudinal perspective and the triangulation of the present self-report scales with observational measures or expert judgements where feasible. Another limitation is that the socio-demographic variables did not include details of the cause of death, obscuring possible variations between, for example, violent vs. natural death bereavement. A final limitation is that we tested the ISLES and ICSG in the same sample. Future research should also establish their psychometric properties in different samples to replicate and extend the generalisation of these results.

## Figures and Tables

**Table 1:** Results of Confirmatory Factor Analysis (*n* = 348).

Scale	Model	Chi-Square ^1^	df	CFI	TLI	RMSEA	Delta Chi-Square	Delta df	*p*-Value	Delta CFI
ICSG	1-factor	1068.762	135	0.978	0.975	0.141	338.025	1	<0.001	−0.008
	2-factors	730.737	134	0.986	0.984	0.113				
ISLES	1-factor	385.035	104	0.991	0.989	0.088	84.29	1	<0.001	−0.003
	2-factors	300.745	103	0.994	0.992	0.074				

Note. CFI = Comparative Fit Index; TLI = Tucker–Lewis Index; RMSEA = Root Mean Square Error of Approximation. ^1^ All chi-square values are significant (*p* < 0.001).

**Table 2:** Descriptive statistics and bivariate correlations for study variables included in path model (*n* = 348).

Variable	M	SD	Range	1	2	3	4	5	6	7	8
1.GMRI_CB	3.85	0.62	1–5	-							
2.GMRI_PG	3.1	0.67	1–5	0.43 *	-						
3.GMRI_SOP	3.08	0.97	1–5	0.11	0.04	-					
4.GMRI_EM	2.67	0.78	1–5	0.23 *	0.20 *	−0.31 *	-				
5.GMRI_VL	3.65	0.71	1–5	0.34 *	0.64 *	0.06	0.13	-			
6.TDRS	18.43	6.44	6–30	−0.44 *	−0.12	−0.09	0.02	−0.12	-		
7.ICSG	22.77	18.15	0–72	−0.25 *	−0.08	−0.26 *	0.26 *	−0.01	0.43 *	-	
8.ISLES	61.66	11.72	16–80	−0.11	−0.14	0.38 *	−0.70 *	−0.11	−0.03	−0.42 *	-

* *p* < 0.05 with Bonferroni adjustment for multiple comparisons.

## Data Availability

Data supporting reported results can be found in the University of Padua Research Data Repository at the following link: http://researchdata.cab.unipd.it/544/ (accessed on 26 October 2021).

## Appendix A

**Italian version of Integration Stressful Life Events Scale and Inventory of Complicated Spiritual Grief**

**Scala per l’Integrazione degli Eventi Stressanti nella Vita (Integration Stressful Life Events Scale)**

Gentile partecipante, le chiediamo adesso di leggere attentamente le seguenti affermazioni che fanno riferimento all’evento della perdita della persona a lei cara. Indichi il grado di accordo per ciascuna di esse queste con un punteggio che va da 1, che significa “Fortemente in disaccordo”, a 5, che significa “Fortemente in accordo”.

1. Da quando è successo questo evento, il mondo sembra un posto disorientante e spaventoso.	1	2	3	4	5
2. Ho dato un senso a questo evento.	1	2	3	4	5
3. Se o quando parlo di questo evento, credo che le persone mi vedano in modo diverso.	1	2	3	4	5
4. Ho difficoltà ad integrare questo evento nella mia comprensione del mondo.	1	2	3	4	5
5. Da quando è successo questo evento, mi sento come se fossi in una crisi di fede.	1	2	3	4	5
6. Questo evento è incomprensibile per me.	1	2	3	4	5
7. I miei precedenti obiettivi e le mie speranze per il futuro non hanno più senso da quanto è successo questo evento.	1	2	3	4	5
8. Sono perplesso per quello che è successo.	1	2	3	4	5
9. Da quando è successo questo evento, non so cosa farò in futuro nella mia vita.	1	2	3	4	5
10. Sarebbe stato più facile parlare della mia vita se avessi omesso questo evento.	1	2	3	4	5
11. Le mie credenze e i miei valori sono meno chiari da quando è successo questo evento.	1	2	3	4	5
12. Non capisco più me stesso da quando è successo questo evento.	1	2	3	4	5
13. Da quando è successo questo evento, ho più difficoltà a sentirmi parte di qualcosa più grande di me.	1	2	3	4	5
14. Questo evento mi ha fatto sentire meno determinato.	1	2	3	4	5
15. Non sono stato in grado di rimettere insieme i pezzi della mia vita da quando è successo questo evento.	1	2	3	4	5
16. Dopo questo evento, la vita sembra più casuale.	1	2	3	4	5

**Scala ala per il lutto spirituale complicato (Inventory of Complicated Spiritual Grief)**

Gentile partecipante, adesso le chiediamo di leggere attentamente le seguenti affermazioni riguardo il suo rapporto con Dio dopo la perdita della persona cara. Indichi poi quanto per lei ciascuna affermazione è vera con un punteggio che va da 0, che significa “Non affatto vero”, a 4, che significa “Assolutamente vero”.

1. Non capisco perché Dio abbia reso tutto così difficile per me.	0	1	2	3	4
2. Mi sono ritirato dalla mia comunità con altri credenti.	0	1	2	3	4
3. Faccio di tutto per evitare attività spirituali / religiose (ad es. preghiera, adorazione, lettura della Bibbia).	0	1	2	3	4
4. Non mi sento più al sicuro e protetto/a da Dio.	0	1	2	3	4
5. Trovo che le attività spirituali / religiose non siano molto soddisfacenti (ad es. preghiera, adorazione, lettura della Bibbia).	0	1	2	3	4
6. Mi è impossibile pregare.	0	1	2	3	4
7. Faccio fatica ad accettare come un buon Dio possa permettere che accadano cose brutte.	0	1	2	3	4
8. Trovo difficile abbandonare la mia vita a Dio.	0	1	2	3	4
9. Non mi sento confortato/a dalla comunità della Chiesa come prima.	0	1	2	3	4
10. Non posso fare a meno di sentirmi arrabbiato/a con Dio.	0	1	2	3	4
11. Non ho molta voglia di unirmi alla comunità per lodare Dio o glorificarlo.	0	1	2	3	4
12. La forte luce guida della mia fede si è indebolita e mi sento perso.	0	1	2	3	4
13. Sono confuso sul motivo per cui Dio avrebbe permesso che questo accadesse.	0	1	2	3	4
14. Ho perso il desiderio di adorare Dio.	0	1	2	3	4
15. Mi è impossibile adorare Dio.	0	1	2	3	4
16. Sento che la mia perdita sia ingiusta.	0	1	2	3	4
17. Sento l’assenza di Dio più di quanto non senta la presenza di Dio.	0	1	2	3	4
18. Sono un credente fedele, quindi non capisco perché Dio non mi ha protetto.	0	1	2	3	4
